# Evaluation of Biological Equivalence for Generic Tulathromycin Injections in Cattle

**DOI:** 10.3390/ijms242216262

**Published:** 2023-11-13

**Authors:** JeongWoo Kang, HyunYoung Chae, SungHoon Jeong, Rokeya Pervin, Md Akil Hossain

**Affiliations:** 1 Animal Disease Diagnosis Division, Animal and Plant Quarantine Agency (APQA), Ministry of Agriculture, Food and Rural Affairs, 177, Hyeoksin 8-ro, Gimcheon-si 39660, Gyeongsangbuk-do, Republic of Korea; hijach@korea.kr (J.K.); iichy33ii@korea.kr (H.C.); 2 Veterinary Drugs & Biologics Division, Animal and Plant Quarantine Agency (APQA), Ministry of Agriculture, Food and Rural Affairs, 177, Hyeoksin 8-ro, Gimcheon-si 39660, Gyeongsangbuk-do, Republic of Korea; bara5239@daum.net; 3 Department of Microbiology and Immunology, College of Medicine, University of Illinois Chicago, 835 S. Wolcott Ave (MC790), Chicago, IL 60612, USA; rpervin@uic.edu; 4 Institute for Tuberculosis Research, College of Pharmacy, University of Illinois Chicago, 833 S. Wood St. (MC964), Chicago, IL 60612, USA

**Keywords:** pharmacokinetic study, bioequivalence, tulathromycin injection, cattle, antibiotics

## Abstract

The recent expiration of patents for the antibiotic tulathromycin has led to a significant increase in the number of generic tulathromycin products (GTPs) available. This study aims to evaluate the bioequivalence of four GTPs, which experienced a rapid increase in market share. The bioequivalence was evaluated by performing pharmacokinetic assessments. The four selected GTPs (Tulaject, Tulagen, Toulashot, and T-raxxin) were compared with the reference product, Draxxin. A dose of 2.5 mg/kg.bw/day was administered via subcutaneous injection, and blood samples were collected 460 times from 20 Holstein cattle. Plasma concentrations of tulathromycin were measured over time using LC-MS/MS analysis. Bioequivalence was evaluated using a statistical program for pharmacokinetic parameters, including the area under the concentration time curve (AUC) and the maximum plasma concentration (C_max_). The bioequivalence was considered proven if the difference between the test and reference products was within 20% for both AUC and C_max_. The results showed that the confidence interval (CI, 90%) for both AUC and C_max_ values was within the 80~120% range, demonstrating the bioequivalence of the four GTPs compared to Draxxin. This study provides evidence for the bioequivalence of the selected GTPs, contributing to their validation for use as effective antibiotics.

## 1. Introduction

Tulathromycin is a semi-synthetic, long-acting macrolide antibiotic derived from erythromycin [1]. Since its introduction, it has been extensively utilized in veterinary medicine for treating respiratory infections in cattle and swine due to its broad-spectrum activity against a range of Gram-positive, Gram-negative, and atypical bacteria, as well as mycoplasmas [2,3]. The unique structural features of tulathromycin, such as its three-position acylsugar moiety and 11,12-carbamate group, contribute to its high potency, rapid bactericidal action, and extended duration of activity [4,5]. However, the original drug is known for its high cost, which has limited its accessibility for many veterinary practitioners and livestock producers. Following the patent expiration of tulathromycin, several animal drug manufacturers in Korea have developed and produced generic versions of the antibiotic.

However, the approval and use of generic drugs hinge on their bioequivalence with the innovator product [6]. To approve generic drugs, the Food and Drug Administration (FDA) requires proof of average bioequivalence in drug absorption, which should be provided by conducting the bioavailability and bioequivalence studies. Bioequivalence assessment is considered as a surrogate for clinical evaluation of the therapeutic equivalence of drug products [7]. In the same way, Animal and Plant Quarantine Agency (APQA), which is the regulatory and research organization of veterinary medicines in South Korea approves generic antimicrobials based on the results of bioavailability and bioequivalence studies with the data of some general quality control, in vitro efficacy against the organism, stability and sterility tests. In South Korea, the approval system for generic animal drugs allows for the exemption from certain evaluations, including in vivo bioequivalence studies, after various assessments have been conducted. The choice of antibiotics for animals in South Korea is very limited due to the antimicrobial resistance. Tulathromycin, in the Korean context, had not yet reported resistance and was considered a component associated with various inconveniences in clinical settings due to its high price. The generic Tulathromycin injections were preliminary approved by APQA based on the quality control, efficacy against the organism, stability and sterility test data at the GLP level provided by the manufacturing companies. These manufacturers were initially waived for bioequivalence studies; thus, they did not conduct the bioequivalent study.

The emergence of generic tulathromycin products has increased their availability in the market, offering cost-effective alternatives to their branded counterparts with similar efficacy and safety profiles [8]. This has the potential to broaden the accessibility of tulathromycin for veterinary use, particularly in the treatment of bovine respiratory disease (BRD) and swine respiratory disease (SRD), which are significant causes of morbidity and mortality in the livestock industry [9]. However, the development of resistance to antibiotics is a growing public health concern, and the prudent use of antimicrobial agents in veterinary medicine is crucial to minimize the risk of resistance development [10]. Ensuring that generic products are bioequivalent to the reference product is essential to maintain their efficacy and prevent the potential emergence of resistant bacterial strains [11]. Bioequivalence studies ensure that the generic product provides the same therapeutic effect as the reference product, which is particularly important for antibiotics such as tulathromycin, given the global concern over antimicrobial resistance and the need for effective treatments [12].

Bioequivalence is typically assessed by comparing the pharmacokinetic parameters, such as the area under the plasma concentration-time curve (AUC) and maximum plasma concentration (C_max_), between the generic and reference products [13]. A generic product is considered bioequivalent to the reference product if the 90% confidence interval (CI) of the ratio of the test to reference product AUC and C_max_ values falls within the predefined limits (usually 80–125%) [13,14]. To comply with the approval process of veterinary drugs in Korea and for post-marketing quality control, the Korean regulatory agency, APQP, conducted in vivo bioequivalence studies of these generic tulathromycin injections. Chemical structures of the active ingredients and the compositions of the innovator product and generic products are mentioned in “Supplementary Information” section. We assessed the pharmacokinetic parameters, including AUC, C_max_, T_max_ and T_1/2_ following a single subcutaneous injection of 2.5 mg/kg bw/day in cattle. Our findings will contribute to the current understanding of the bioequivalence of generic tulathromycin products and provide valuable information for veterinarians and livestock producers in selecting cost-effective and therapeutically equivalent alternatives to the reference product. Additionally, our study will aid regulatory authorities in evaluating the safety and efficacy of generic tulathromycin products, ultimately promoting their responsible use in the livestock industry.

## 2. Results

### 2.1. Potency of Generic Products

**Figure 1:**
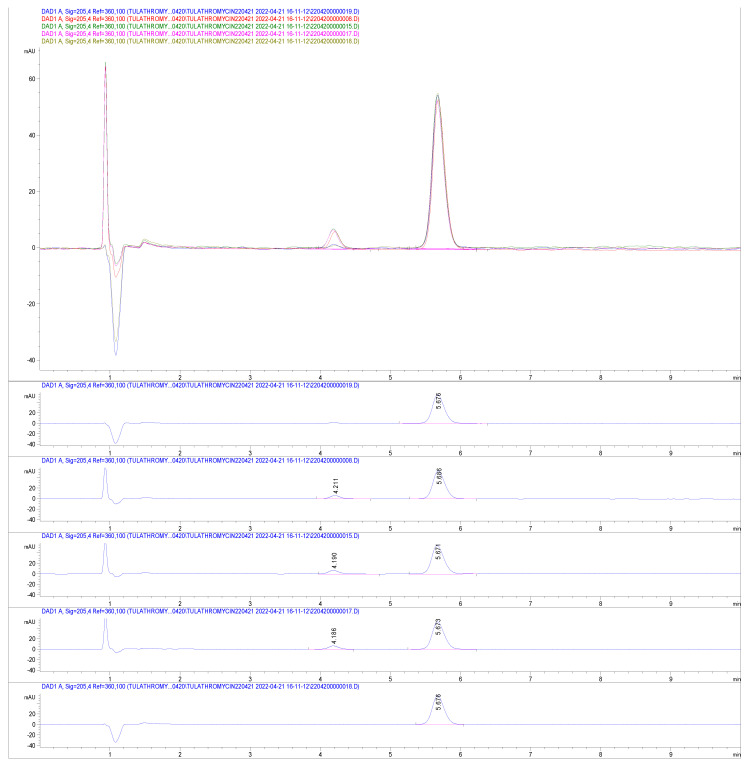
Chromatograms of tulathromycin in innovator drug (Draxxin), and generic drugs (Tulaject, Tulazen, Tulshot, and T-raxxin) by HPLC.

Potencies of generic products compared to their label claim were examined by HPLC. The chromatograms show the peak of tulathromycin in generic products from various sources (Figure 1). The retention time and area of tulathromycin peak from four tested generic products were almost the same as these found in the reference tulathromycin injection. No interference with the tulathromycin peak was found from the mobile phase and excipients of those products. Comparative potencies of tulathromycin generic products are shown in Table 1. Potency found for Draxxin, Tulaject, Tulagen, Toulashot, and T-raxxin were 101, 98, 95, 100, 97 mg/mL, respectively. These results confirm the similarity and acceptability of those batches of generic products to be used for bioequivalence study. Thus, from the same batch, the products were administered to cattle for the bioequivalence study.

### 2.2. Validation of LC-MS/MS Method

**Figure 2:**
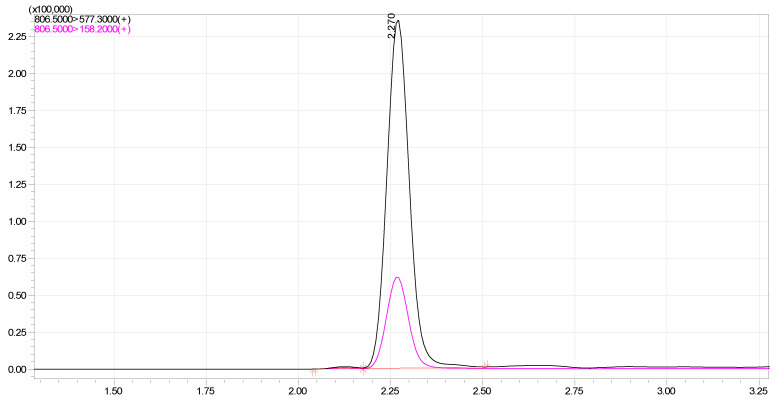
Mass chromatogram of Tulathromycin (precursor and daughter ion) in blood sample of cattle.

A liquid chromatography with the tandem mass spectrometry (LC–MS/MS) method was used to determine the amount of tulathromycin A and B in the plasma of cattle. Tulathromycin-spiked plasma samples were used to validate the method prior in order to apply this method in sample analysis. It is clear from the chromatograms (Figure 2) of the developed method that there are no noticeable interferences from the matrix and the analyte. This, in turn, ensures obtaining reliable results with this method for determining the biological concentrations of tulathromycin. Under expressed chromatographic conditions, the retention time was approximately 2.27 min for tulathromycin. This method provided linear responses throughout the concentration range, which is suitable for the intended purposes of this study. The correlation coefficient (R^2^), limit of detection (LOD) and limit of quantitation (LOQ) values of this method were 0.9961, 0.23 ng/mL and 0.76 ng/mL, respectively. The mean recovery of tulathromycin in this method was within the range of 87.4–91.6%, precision and accuracy for the 1, 2, 5, 25, and 50 μg/mL of tulathromycin, which were all within 3.7–9.8% in plasma. These data clearly show that the developed method has an acceptable degree of repeatability and accuracy within an analytical run.

### 2.3. Pharmacokinetic Profile

**Figure 3:**
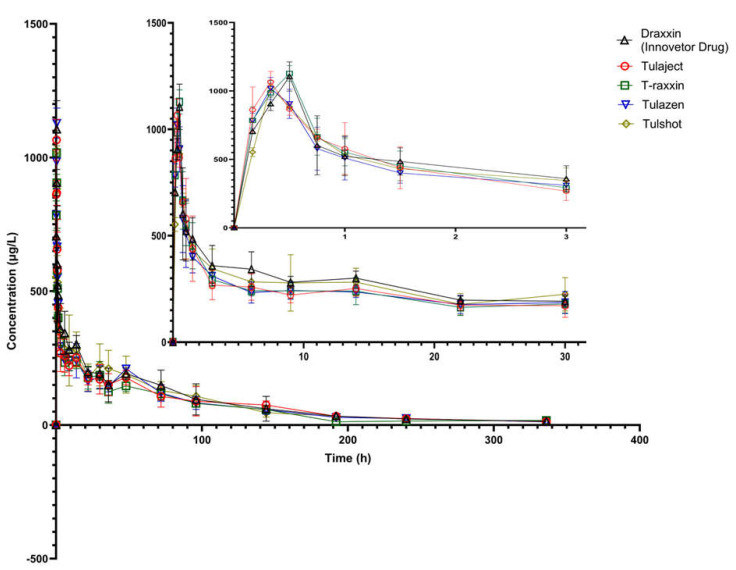
Comparative plasma concentration versus time curves of tulathromycin following injection of innovator or generic drugs (2.5 mg/kg) to cattle. Each time point represents the drug concentration in Mean ± SD (n = 4).

Holstein breed 20 cattle were divided into five groups where four animals were assigned in each group for pharmacokinetic analysis. After one subcutaneous injection with a dose of 2.5 mg/kg body weight, blood samples were collected from each animal. The collected blood samples were analyzed using LC–MS/MS. The mean plasma concentration-time profiles after single-dose subcutaneous injection of the tulathromycin inventor drug and four generic drugs are shown in Figure 3. The mean plasma concentration-time curves from the four test and one inventor products were almost superimposed. Moreover, it was found that there was no significant difference of plasma tulathromycin concentrations from generic products compared to innovator’s product at each time points. Further, a quantifiable amount of drug was found at the first case-time (10 min), in almost all subjects following the administration of each product. The pharmacokinetic profiles such as T_1/2_, T_max_, C_max_, and AUC of the tulathromycin innovator drug and generic drugs are shown in Table 2. The C_max_ of tulathromycin from Draxxin (Zoetis, first approved product; reference drug), Tulaject (Thumbvet), Tulazen (Hanawin), Tulshot (Eaglevet), and T-raxxin (Komipharm) injections were 1100.08 ± 102.97, 1027.97 ± 77.06, 1110.04 ± 56.78, 1031.15 ± 80.09 and 1028.99 ± 18.95 ng/mL, respectively. The time required to reach the maximum plasma concentration (T_max_) of tulathromycin from Draxxin (reference drug), Tulaject, Tulazen, Tulshot, and T-raxxin (4 generic drugs) were 0.44 ± 0.08, 0.33 ± 0.00, 0.50 ± 0.00, 0.33 ± 0.09 and 0.33 ± 0.00 h, respectively.

### 2.4. Bioequivalence Analysis

The AUC and C_max_ were considered as the key parameters for determining the bioequivalence of the tulathromycin innovator product and generic products. The bioequivalence was evaluated by determining 90% confidence intervals of the C_max_ and AUC. The 90% confidence interval values of the tulathromycin generic product Tulaject, Tulazen, Tulshot, and T-raxxin considering AUC were (84.04–115.95)%, (90.43–109.59)%, (89.07–110.96)%, and (82.19–117.83)%, respectively, which are within the bioequivalence range (80–120)% of the reference product (85.19–114.80)%. The 90% confidence interval values of the tulathromycin generic product Tulaject, Tulazen, Tulshot, and T-raxxin in C_max_ were (93.84–106.13)%, (95.76–104.23)%, (93.61–106.39)%, (98.45–101.56)%, respectively, which were also within the bioequivalence range (80–120)% of the innovator product (Table 3).

## 3. Discussion

Antibiotics are antimicrobial compounds that are used in both human medicine and animal agriculture to reduce incidences of diseases [15]. The efficacy of the drug is characterized by a rapid rate of absorption, high bioavailability, extensive tissue distribution, and longer elimination half-lives in the plasma and lungs of animal [16,17]. The supply of the drug in reliable and quality standards is very important for the efficacy of the drug as well as for the health of treated subjects. Otherwise, substandard medicines can cause poisoning, untreated disease, early death, treatment failure, and an increase in antimicrobial resistance problems [18]. Before marketing a brand-name drug, the inventing company of that drug typically studies the intended use, safety, efficacy, strength, route of administration, best dosage form, quality, and performance characteristics. A generic drug is created to be the same as an already marketed brand-name drug. These similarities help to demonstrate bioequivalence, which means that a generic medicine provides the same clinical benefit and works in the same way as the brand-name medicine. The uses of tulathromycin in veterinary practice are very wide. However, the original drug is known for its high cost, which has limited its accessibility for many veterinary practitioners and livestock producers, and the generic drug obtained a large market share.

The generic tulathromycin injections were preliminary approved in Korea by APQA based on the quality control, efficacy against the organism, and stability and sterility test data at the GLP level, provided by the manufacturing companies. These manufacturers were initially waived for bioequivalence studies. To comply with the approval process of veterinary drugs in Korea and for post-marketing quality control, the Korean regulatory agency, APQP, conducted in vivo bioequivalence studies of these generic tulathromycin injections. The bioequivalence analyses of four tulathromycin generic medicines were performed in this study following the recommendations in the “*Bioequivalence Study Guide for Veterinary Medicinal Products*” published by the European Medicines Agency [19]. The pharmacokinetic properties (C_max_, AUC, T_max_, and T_1/2_) of those generic medicines were selected as primary pharmacokinetic parameters and were evaluated to determine the bioequivalence.

In this study, single dose of tulathromycin subcutaneous injections were administered to cattle and it was found that all four generic products were well-tolerated by all subjects, with no clinical adverse events. The analytical method showed good specificity, linearity, accuracy, and precision for the quantitation of tulathromycin in plasma samples, thus allowing its use in bioequivalence assays. The plasma concentrations of tulathromycin were evaluated at different time points till 336 h after injection to cattle. The plasma concentration profiles of tulathromycin indicate that the generic tulathromycin medications were not significantly different from the brand-name drug when injected in cattle. The C_max_ values of reference drug (Draxxin) and four generic drugs (Tulaject, Tulazen, Tulshot and T-raxxin) were 1100.08 ± 102.97, 1027.97 ± 77.06, 1110.04 ± 56.78, 1031.15 ± 80.09 and 1028.99 ± 18.95 ng/mL, respectively. The MICs of tulathromycin were 0.25 µg/mL and 0.03 µg/mL against *A. pleuropneumoniae* and *P. multocida* in the infection model [20,21]. The C_max_ values of tulathromycin from the reference and generic products were at least four times the MIC (≤0.25 μg/mL) against common microorganisms such as *A. pleuropneumoniae* and *P. multocida*.

The AUC is a useful metric that expresses the total amount of drug that comes into the systemic circulation after drug administration [22]. In our study, the AUC value for the reference product was 27,187.01 ± 4891.20 μg.h/mL, and for four generic products were 27,452.51 ± 5327.37, 26,983.37 ± 3146.46, 26,036.92 ± 3465.66, and 26,962.52 ± 5844.84 μg.h/mL. The results show that there is no such difference in the AUC values of four generic products with the AUC value of the reference product. We also determined the values of other pharmacokinetic parameters such as T_1/2_ and T_max_ for the reference and generic drugs; and found that there were no substantial differences in the parameters among these products.

The 90% confidence interval in the average AUC and C_max_ values among the standard drug (Draxxin injection) and generic products (Tulaject injection, Tulazen injection, Tulshot injection, and T-raxxin injection) are completely within the acceptable bioequivalence range of 80–120% as set by the FDA and European Medicines Evaluation Agency [11]. The results of our study confirm that Draxxin injection is bioequivalent to Tulaject, Tulazen, Tulshot, and T-raxxin injections and can be safely used interchangeably.

## 4. Materials and Methods

### 4.1. Reagents and Chemicals

High-purity tulathromycin from Sigma-Aldrich (St. Louis, MO, USA) was used as a standard. HPLC-grade acetonitrile was purchased from J.T. Baker (Phillipsburg, NJ, USA), and hydrogen chloride solution was obtained from Merck (Darmstadt, Germany). Distilled water from EMD Millipore (Billerica, MA, USA) was used with a Milli-Q Integral Millipak membrane point-of-use cartridge (0.22 μm). Other reagents and solvents used for analysis were of analytical grade or higher. Five veterinary pharmaceutical products such as Draxxin (Zoetis, Parsippany-Troy Hills, NJ, USA; first approved product; control drug), Tulaject (Thumbvet, Iksan-si, South Korea), Tulazen (Hanawin, Jeonnam, South Korea), Tulshot (Eaglevet, Yesan, South Korea), and T-raxxin (Komipharm International Co. Ltd, Seoul, South Korea) injections were collected from retail pharmacy.

### 4.2. High-Performance Liquid Chromatography (HPLC)

These tulathromycin products were analyzed by HPLC to check whether the potencies of these products were (100 ± 5)% to the label claim or not. The products from the same batch/lot number of a manufacturer having 95–105% potency were used in this bioequivalence study. The Agilent Technologies 1260 equipped with a diode array detector (Santa Clara, CA, USA) was used to perform HPLC analysis. To analyze each substance, a Triart C18 column (150 × 4.6 mm, 3 μm) manufactured by YMC America Inc. (Devens, MA, USA) was used. The mobile phase was a mixture of water and acetonitrile (70:30) and the flow rate of the mobile phase was 1 mL/min. The sample injection volume was 10 µL. Compound was detected at a wavelength of 220 nm.

### 4.3. Animal Experiment

Twenty Holstein breed cattle were acclimatized and adapted to the environment for 1 week before commencing experimentation. All the cattle were male (steers), aged around 6 months, and purchased from the CRO company, KULF Co., Ltd. (Namyangju, South Korea). Approximate body weight of each animal was 100 kg, and the animals were divided into five groups where four animals were assigned in each group. After one subcutaneous injection with a dose of 2.5 mg/kg body weight, 460 blood samples were collected per animal at 0, 10 min, 20 min, 30 min, 45 min, 60 min, 1.5 h, 3 h, 6 h, 9 h, 14 h, 22 h, 30 h, 36 h, 48 h, 72 h, 96 h, 144 h, 192 h, 240 h, and 336 h. After collecting blood samples, the plasma was separated and frozen at −80 °C for blood concentration measurements. The collected blood samples were analyzed using liquid chromatography with tandem mass spectrometry (LC–MS/MS). During these 14 days of blood sampling, clinical symptoms were monitored, and blood and antigen tests for potential infectious diseases were performed when necessary. This animal experiment was conducted at KULF Co. Ltd., which is equipped with specialized animal care and management facilities adequate for performing pharmacokinetic and biological equivalence tests. The animal experimental protocol was approved by the Institutional Animal Care and Use Committee (IACUC, Approval number 2022-660).

### 4.4. LC–MS/MS Analysis Conditions

To determine the amount of tulathromycin A and B in the plasma, a YMC C18 (3.0 × 100 mm) column of 3 μm inner porosity was used in an LC–MS/MS-8045 system (Shimadzu, Tokyo, Japan). The mobile phase was a mixture of (A) 0.1% formic acid in acetonitrile and (B) 0.1% formic acid in distilled water. The gradient flow was maintained with the flowrate of 0.6 mL/min. The mobile phase was initially allowed to flow with a 10:90 ratio of A:B phases. From 0.1 to 3 min, the ratio of mobile phase solvents was gradually altered to 100% of “A” and 0% of “B” phases, and from 3.1 min, these proportions were maintained until 3.9 min. The ratio was altered to 95:5 of “A” and “B” phases from 4 to 4.9 min. The proportion of the mobile phase solvents were then reverted to the initial ratio (10% “A” and 90% “B”) at 5 min, and this composition was used until the end of the acquisition. Five µL of the sample was injected at each time. The method was optimized and validated prior to undertaking PK analysis. The mass spectrometer conditions were optimized using electrospray ionization (ESI) in the positive ion mode.

### 4.5. Validation of LC–MS/MS Method

The specificity of the tulathromycin quantification method was evaluated by injecting tulathromycin standard solution and tulathromycin-untreated plasma spiked with known concentrations of tulathromycin; this helped in determining whether the matrix showed any interference at the retention time of tulathromycin. The tulathromycin stock solution (1 mg/mL) was prepared by dissolving the compound in 0.1% aqueous formic acid. The pH of the stock solution was stabilized to establish linearity. A series of standard enrofloxacin solutions were made by further diluting the stock solution. Various concentrations of tulathromycin solution were added to plasma of the untreated chicken to prepare different concentrations (1, 2, 5, 25, and 50 ng/mL) of tulathromycin-spiked plasma samples. These samples were injected to LC–MS/MS and the tulathromycin concentrations were determined. Recommendation of tulathromycin elevated dose in cattle in the plasma matrix were quantified. The concentration of tulathromycin obtained from different samples using LC–MS/MS analysis was used to calculate accuracy, linearity, recovery percentage, calibration curve, and regression coefficient values. Three different concentrations of spiked plasma samples were injected six times individually to examine repeatability and reproducibility. The limit of detection (LOD) and limit of quantitation (LOQ) were calculated from the calibration curve by analyzing tulathromycin-spiked samples. The LOD and LOQ were determined from the slope of the calibration curve and the standard deviation (SD) of the responses were calculated using the following equations: LOD = (3.3 × SD)/slope and LOQ = (10 × SD)/slope [23].

### 4.6. Pharmacokinetic and Bioequivalence Analysis

The concentration of tulathromycin in the plasma samples of cattle was quantified by LC–MS/MS. Different features of the pharmacokinetics of tulathromycin were determined using WinNonlin software version 6.1 (Pharsight Corporation, Mountain View, CA, USA). The elimination half-life (T_1/2_), area under the plasma concentration–time curve (AUC), concentration maxima (C_max_) and peak time to reach the C_max_ (T_max_) were estimated by using non-compartmental analysis. The C_max_ and AUC were considered as the key factors for determining the bioequivalence of the innovator product and four generic products of tulathromycin. The bioequivalence was evaluated by determining 90% confidence intervals of the innovator drug and four generic drugs of tulathromycin.

### 4.7. Statistical Analysis

Results are presented as the mean ± SD of triplicate examinations. F-test and one-way analysis of variance were used for statistical analyses. Statistical significance was considered present if *p*-values were less than 0.05.

## Figures and Tables

**Table 1:** Comparative potency of tulathromycin generic products by HPLC method.

Name	Manufacturer	Label Claim (mg/mL)	Found Potency (mg/mL)	Percent of Label Claim (%)
Draxxin	Zoetis Inc., Parsippany-Troy Hills, NJ, USA (Pioneer Company)	100	101	101
Tulaject	Thumb Vet Co., Ltd., Iksan-si, Korea	100	98	98
Tulagen	Hanawin Co., Ltd., Jeonnam, Korea	100	95	95
Toulashot	Eagle Vet Co., Ltd., Yesan, Korea	100	100	100
T-raxxin	Komipharm International Co., Ltd., Seoul, Korea	100	97	97

**Table 2:** Pharmacokinetic profiles of tulathromycin injection from the innovator product and 4 generic products.

Parameters (Unit)	Inventor Drug	Tulaject Injection (Thumbvet)	Tulazen Injection (Hanawin)	T-raxxin Injection (Komipharm)	Tulshot Injection (Eaglevet)
T_1/2_ (h)	94.04 ± 36.09	102.56 ± 24.31	91.40 ± 6.13	96.17 ± 14.07	96.79 ± 36.43
T_max_ (h)	0.44 ± 0.08	0.33 ± 0.00	0.50 ± 0.00	0.33 ± 0.09	0.33 ± 00
C_max_ (ng/mL)	1100.08 ± 102.97	1027.97 ± 77.06	1110.04 ± 56.78	1031.15 ± 80.09	1028.99 ± 18.95
AUC (hr.ng/mL)	27,187.01 ± 4891.20	27,452.51 ± 5327.37	26,983.37 ± 3146.46	26,036.92 ± 3465.66	26,962.52 ± 5844.84

T_1/2_, the elimination half-life. T_max_, time to reach maximum plasma concentration. AUC, area under the plasma concentration-time curve. C_max_, maximum plasma concentration.

**Table 3:** The 90% Confidence Interval of reference and generic products of tulathromycin injection.

Parameter	Inventor Drug	Tulaject Injection (Thumbvet)	Tulazen Injection (Hanawin)	T-raxxin Injection (Komipharm)	Tulshot Injection (Eaglevet)
C_max_	1015.00–1185.00	964.60–1091.00	1063.00–1157.00	965.30–1097.00	1013.00–1045.00
	92.27–107.72%	93.84–106.13%	95.76–104.23%	93.61–106.39%	98.45–101.56%
AUC	23,160.00–31,210.00	23,070.00–31,830.00	24,400.00–29,570.00	23,190.00–28,890.00	22,160.00–31,770.00
	85.19–114.80%	84.04–115.95%	90.43–109.59%	89.07–110.96%	82.19–117.83%

C_max_, maximum plasma concentration. AUC, area under the plasma concentration-time curve.

## Data Availability

The data presented in this study are available in the article and Supplementary Information.

## Supplementary Materials

The following supporting information can be downloaded at: https://www.mdpi.com/article/10.3390/ijms242216262/s1.
