# Supplementary material for: Evaluation of Biological Equivalence for Generic Tulathromycin Injections in Cattle
Source: Int J Mol Sci. 2023 Nov 13;24(22):16262. doi: 10.3390/ijms242216262 (PMC10671567; doi:10.3390/ijms242216262)
Supplement: Supplementary file 1 [file ijms-24-16262-s001.zip › ijms-2673772-supplementary.pdf]

Supplementary Information

The original drug, Draxxin, and its SPC (Summary of Product Characteristics) indicate that the active ingredient (tulathromycin) and the excipients' content are identical in all the generic products. At the time of approval, products with the same ingredients, content, administration method, and dosage were granted sales authorization. Compositions of the innovator and generic tulathromycin injections are presented below.

<Draxxin>

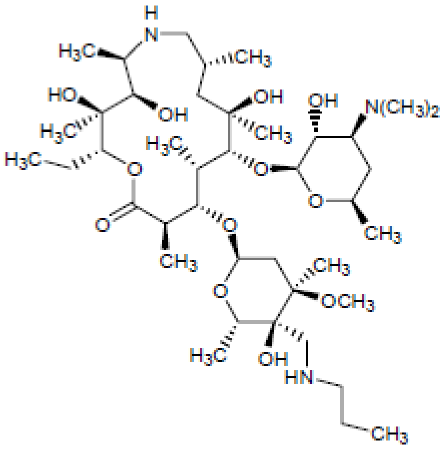

CP-472,295

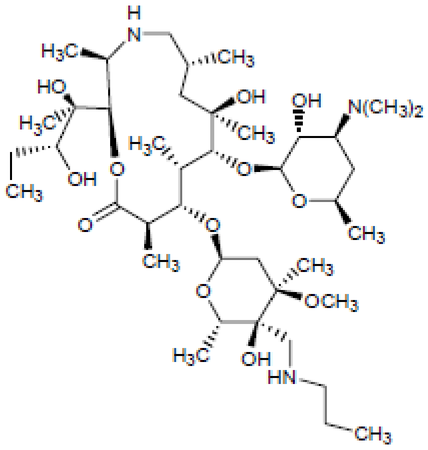

CP-547,272

Ingredients	Amount in 1 mL of Drug
Tulathromycin	100mg
Monothioglycerin	5.0mg
Anhydrous Citric Acid	19.2mg
Hydrochloric Acid	19.0mg
Sodium Hydroxide	Appropriate amount
Propylene Glycol	500mg
Water for Injection	Appropriate amount

<Generics>

Tulathromycin A

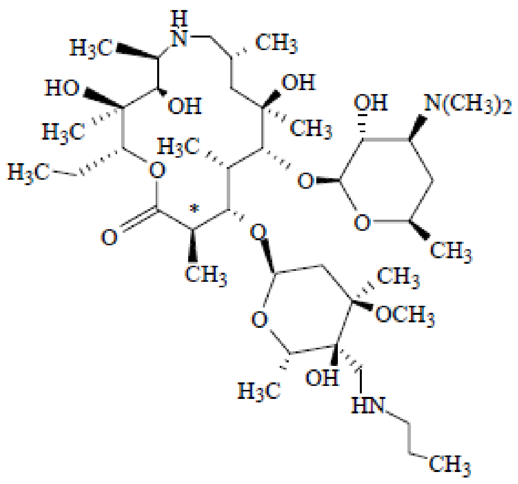

Tulathromycin B

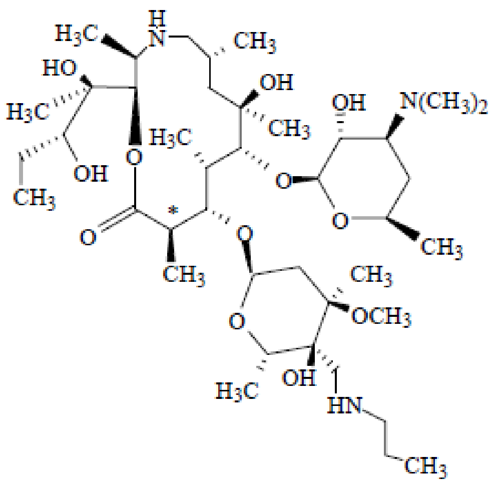

**Ingredients**	**Amount in 1 mL of Drug**
Tulathromycin	100mg
Monothioglycerin	5.0mg
Anhydrous Citric Acid	19.2mg
Hydrochloric Acid	19.0mg
Sodium Hydroxide	Appropriate amount
Propylene Glycol	500mg
Water for Injection	Appropriate amount
